# Supplementary material for: MetaRibo-Seq measures translation in microbiomes
Source: Nat Commun. 2020 Jun 29;11:3268. doi: 10.1038/s41467-020-17081-z (PMC7324362; doi:10.1038/s41467-020-17081-z)
Supplement: Supplementary file 10 — Supplementary Data 7 [file 41467_2020_17081_MOESM10_ESM.zip › File2/Confidence_High_Taxonomy/236927_out.krona.html]

Javascript must be enabled to view this page.

members
magnitude
magnitudeUnassigned
count
unassigned
taxon
rank

236927\_out

2

2
superkingdom
2

1239
phylum
2

1
class
186801

186802
1
order

1
family
541000

genus
1
292632

species
1

SRS143895\_contig\_number\_16499
1897022

91061
class
1

1
order
186826

family
1
1300

1
genus
1301

species
1

SRS142787\_contig\_number\_contig-100\_12744.12744
1304
